# Prospective Study on the Evaluation of Echocardiographic Parameters as Predictors of a Positive Response to Cardiac Resynchronization Therapy in a Tertiary Care Hospital in Mexico

**DOI:** 10.3390/jcm15020609

**Published:** 2026-01-12

**Authors:** Juan Carlos Plata-Corona, Karla Sofia Chávez-Gómez, Enrique Torres-Rasgado, Heberto Aquino-Bruno, José Omar Arenas-Díaz, Elias Terrazas-Cervantes, Nilda Espinola-Zavaleta

**Affiliations:** 1 Centro Médico Nacional “Manuel Ávila Camacho”, Instituto Mexicano del Seguro Social, Calle 2 Norte, Puebla 72089, Mexico; 2 Facultad de Medicina, Universidad de las Américas Puebla, Ex Hacienda Santa Catarina, Puebla 72810, Mexico; 3 Facultad de Medicina, Benemérita Universidad Autόnoma de Puebla, Calle 13 Sur 2702, Colonia Los Volcanes, Puebla 72420, Mexico; 4 Hospital General de México “Eduardo Liceaga”, Calle Dr. Balmis 148, Col. Doctores, Ciudad de México 06720, Mexico; 5 Centro Médico Nacional “La Raza”, Instituto Mexicano del Seguro Social, Ciudad de México 06600, Mexico; 6 Sociedad Cardiovascular SC, Mexicali 21100, Mexico; 7 Instituto Nacional de Cardiología “Ignacio Chávez”, Juan Badiano #1, Tlalpan, Ciudad de México 14080, Mexico

**Keywords:** left ventricular ejection fraction, cardiac resynchronization therapy, heart failure, dyssynchrony parameters, left ventricular end-systolic volume

## Abstract

**Background/Objectives**: Heart failure is a major global health problem. Among the available treatment options, cardiac resynchronization therapy (CRT) has been shown to improve both quality of life (QoL) and mortality; however, not all patients respond adequately. Our study aimed to identify echocardiographic parameters that predict a positive response to CRT. **Methods**: A total of 33 patients (10 women and 23 men) were prospectively recruited, all met the standard criteria for CRT implantation. Biochemical, clinical, QoL, 6 min walk test, and echocardiographic evaluations were performed prior to CRT implantation and reassessed after 6 months. A ≥15% reduction in left ventricular end-systolic volume was taken as the defining parameter of positive response. Based on response level, patients were divided into two groups: responders and non-responders. **Results**: Comparing the overall population before and after CRT, a positive impact was observed on biochemical, electrocardiographic, and echocardiographic parameters. Fourteen patients (42%) were classified as responders and nineteen (58%) as non-responders. Only two basal echocardiographic parameters showed significant baseline differences between groups: Global Longitudinal Strain (GLS) and the Kapetanakis index. ROC curve analysis showed that baseline GLS and Kapetanakis index had excellent discriminative ability for predicting CRT response. Also, binary logistic regression analysis identified the association of GLS and Kapetanakis index with CRT response. Finally, Rho Spearman analysis showed a positive correlation between the degree of response to CRT and the QoL, (ρ) of 0.663 with *p* = 0.001. **Conclusions**: Our findings confirm the overall clinical, biochemical, echocardiographic, and QoL benefits of CRT. In addition, two echocardiographic parameters proved to be potential response predictors.

## 1. Introduction

Heart failure (HF) can be defined as inadequate cardiac output that fails to meet metabolic demands, or as adequate output that is maintained only through compensatory neurohormonal activation, which typically manifests as elevated left ventricular (LV) diastolic pressures and increased cardiac biomarkers [1]. Due to its rising prevalence, HF has become a major health problem [2].

According to the Framingham Heart Study, the mortality rate after an HF diagnosis in the United States is approximately 10% at 30 days, 20–30% at 1 year, and 45–60% at 5 years of follow-up [3]. Current evidence in HF with reduced left ventricular ejection fraction (LVEF) has shown that new pharmacological agents and devices, such as implantable cardioverter-defibrillators, and cardiac resynchronization therapy (CRT), significantly improve clinical outcomes in patients with this condition [4].

The concept of CRT is based on the fact that in patients with HF it is common to observe electrical conduction delays, with 25–50% of patients presenting a QRS duration > 120 milliseconds (ms) and 15–27% showing complete left bundle branch block (LBBB). In addition, in these patients, auriculoventricular (AV) synchrony is also often altered, with a prolonged PR interval in up to 52% of cases [5,6]. These electrical disturbances lead to AV, interventricular, and intraventricular dyssynchrony, which eventually results in a decrease in LVEF [7].

According to current international guidelines, device implantation is indicated in patients with HF with LVEF ≤ 35%, wide QRS complexes (≥150 milliseconds) with LBBB morphology, sinus rhythm, and NYHA functional class II–IV despite optimal medical therapy [8]. Nevertheless, several trials showed that, even with appropriate selection criteria, a large proportion of patients do not respond favorably to this therapy [9,10]. Considering that CRT is a high-cost therapy not exempt from complications, attention has focused on identifying variables capable of predicting the response to CRT prior to its implementation.

Information regarding CRT in the Latin American population remains scarce. More recently, in 2023, a retrospective Mexican registry was published [11]. This study analyzed data from patients with HF and reduced LVEF who underwent CRT implantation over a 10-year period, providing valuable insights and highlighting differences compared to international data, mainly in epidemiology, baseline patient characteristics, and cardiovascular outcomes. Based on this information, it was reasonable to consider that the current benefit of CRT might not apply equally to all populations.

Recent publications suggest that there is evidence supporting the use of echocardiographic parameters to improve characterization outcomes, especially in the gray zone of patients with unfavorable electric characteristic, such as intermediate QRS duration, in whom the benefit of CRT remains uncertain [12].

Echocardiographic dyssynchrony parameters have a strong pathophysiologic rationale and were shown to be robust predictors of CRT response in recent observational and retrospective studies [13,14].

Even with this information, other studies and current guidelines consider that its clinical utility is limited and do not recommend its exclusive use for CRT indication due to its variability and lack of validation in large randomized trials [15,16].

Given the limited prospective information available on HF patients undergoing CRT in the Latin American population, the aim of the present study was to identify echocardiographic variables capable of predicting a favorable response to CRT and, in turn, to evaluate the degree of response to the therapy, as well as to characterize and compare the population in accordance with the current literature.

## 2. Materials and Methods

**Study Population:** The study was carried out in the National Institute of Cardiology in Mexico City.

**The inclusion criteria were as follows:**

-Both genders, over 18-year-old patients.

-Diagnosed with HF with reduced LVEF, considered good candidates for CRT according to current guidelines.

-Patients who agreed to sign informed consent to participate in the study and committed to 6-month follow-up.

-Patients in whom the 6 min walk test (6MWT), laboratory tests, echocardiogram and Kansas City Cardiomyopathy Questionnaire (KCCQ) could be performed prior to device implantation and at 6 months post-implantation.

**The exclusion criteria were as follows:**

-Patients with prior atrial fibrillation and/or flutter.

-Patients with a previous pacemaker.

-Acute decompensated congestive HF.

-Patients with limitations that prevent the performance of the required studies (walking test, laboratory tests, QoL questionnaire).

**Elimination criteria:**

-Patients in whom the CRT could not be implanted.

-Loss to 6-month follow-up or absence of complete medical records and data.

The protocol, informed consent, study procedures, and measurements were approved by the bioethics committee of hospitals involved. Prior written informed consent was obtained from each patient to be included in the study. We certify that the study was performed in accordance with the ethical standards as laid down in the 1964 Declaration of Helsinki and its later amendments or comparable ethical standards.

**Study design and protocol:**

An analytical, observational, longitudinal, prospective study was designed.

Due to the high cost of therapy and, consequently, the limited number of patients who receive it, the initial sample size was determined by the total number of patients who met the inclusion criteria and underwent CRT implantation during the period from 2020 to 2024 (n = 42). A total of 9 patients were excluded: 8 did not attend the 6-month follow-up, and in 1 patient, CRT implantation was unsuccessful. The final total population included in this study was 33 patients admitted between March 2020 and April 2024.

Patients were evaluated before and after CRT implantation following the steps below:

1. Patient identification, collection of general information, anthropometric data, and clinical history.
2. QoL evaluation by the application of the KCCQ, results assessed the following domains: physical and social limitation, frequency of symptoms, QoL, and overall total score.
3. Collection of general laboratory tests (complete blood count, chemistry panel, electrolytes, NT-proBNP, Troponin) through venipuncture with the obtention of 20 mL of blood for immediate processing in the institute’s laboratory.
4. Transthoracic echocardiogram evaluation by two experienced echocardiographers. The evaluation was performed in M-mode, 2D, 3D, and Doppler. Images were obtained with a 3.5 MHz transducer at appropriate depth in parasternal (long- and short-axis) and apical views. LV end-systolic volume (LVESV), LV end-diastolic volume, and LVEF were calculated using the biplane Simpson method. As this is an operator-dependent study all echocardiographic studies were independently reviewed by two experienced echocardiographers who were blinded to clinical outcomes, and final measurements were obtained by mutual agreement.
5. 6MWT was performed in a 30 m hallway/corridor, with baseline and per-lap measurements of pulse oximetry and heart rate. Symptoms were described using the Borg scale, oxygen saturation, heart rate, and blood pressure recorded at baseline, at the end of the test, and at 1, 3, and 5 min after completion.
6. Steps 2, 3, 4, and 5 were repeated at 6 months after CRT implantation.

Patients were divided in two groups (responders and non-responders) according to their response to CRT. A positive response was defined as a ≥15% reduction in LVESV at 6 months after CRT implantation.

The primary endpoint was to identify basal (pre-CRT) echocardiographic variables that could predict a positive response to CRT before implantation.

Secondary endpoints included the evaluation of improvements in the KCCQ score and the 6MWT, as well as the correlation between these improvements and the level of response to CRT.

**Echocardiographic parameters:** The following variables were used to assess echocardiographic dyssynchrony, with their respective cutoff values.

1. **Auriculo-ventricular dyssynchrony parameters**
  ‑ Ventricular filling time: The time from mitral valve opening (start of filling) to closure (end of filling) and the relation (<40%) with the R-R interval.
2. **Intraventricular dyssynchrony parameters**
  ‑ Septal–Lateral Delay: Difference between time-to-peak systolic velocity (Ts) of septal and lateral walls with tissue Doppler. Dyssynchrony was defined as Ts ≥ 65 ms.
  ‑ Septal/posterior wall contraction difference in M-mode (ms): Difference in M-mode between the septal and posterior wall greater than 130 ms.
  ‑ Apical rocking: side-to-side motion of the apex from early lateral activation.
  ‑ Septal flash: early septal contraction followed by stretch; strongly associated with CRT response.
  ‑ Systolic Dyssynchrony Index (SDI)/Kapetanakis Index: SD of the time to minimal systolic volume in 16 segments. Abnormal usually > 10% of the cardiac cycle.
3. **Interventricular dyssynchrony parameters**
  ‑ Difference between LV pre-ejection time and RV pre-ejection time: Interval from QRS onset to the onset of aortic and pulmonary flow (measured by pulse-wave Doppler in the LV and RV outflow tract). Difference greater than 50 ms between pre-ejection periods was consider as a dyssynchrony parameter.

**Procedural Details:** All procedures were performed using quadripolar LV leads. The preferred lead position was the posterolateral vein at the basal-to-mid segment; the second option was the anterolateral vein at the basal-to-mid segment. LV lead pacing thresholds were generally <2.5 V at 0.4 ms. The percentage of biventricular pacing was assessed during follow-up device interrogation, with optimization aimed at achieving at least 95%. Device optimization was performed every 6 months using electrocardiographic guidance. The atrioventricular interval was programmed at 120–150 ms. For interventricular (V–V) timing, the initial setting was LV–RV at −30 ms. If QRS duration remained prolonged, sequential adjustments were tested (0, −10, −20, −30, −40, −50, −60, −70, and −80 ms), and the configuration resulting in the narrowest QRS complex was selected.

**Sample Size**

The present study aims to evaluate differences in clinical, electrocardiographic, and echocardiographic variables between heart failure patients who respond or do not respond to CRT, defined as a reduction in LVESV ≥ 15% at six months of follow-up. The main dependent variable is the change in LVESV, measured in milliliters and expressed in percentage. No formal a priori sample size calculation was performed due to the exploratory nature of the study. Sample size was determined by the total number of patients who met the inclusion criteria and underwent CRT implantation. Post hoc considerations based on the Peduzzi rule indicated that only parsimonious logistic regression models could be constructed.

**Statistical analysis**

Statistical analyses were performed using the Statistical Package for the Social Sciences program for Windows, version 25 (SPSS, Chicago, IL, USA).

**Descriptive Analysis:**

Qualitative variables were described as frequencies and proportions. Quantitative variables, in the case of normal distribution, were presented as mean and standard deviation (±/SD), while in the case of non-normal distribution they were presented as median and interquartile range (IQR). Numbers and percentages were used for categorical variables. The Shapiro–Wilks normality test was performed (given the small sample size).

**Inferential Analysis:**

The Chi-square (χ^2^) test was applied to determine the relationship between categorical variables in the general population and in the contrasted populations before and after CRT. For quantitative variables with normal distribution, Student’s *t*-test was applied for related samples. For quantitative variables with non-normal distribution in related groups, the Wilcoxon test was used.

**Discriminative Analysis:**

The discriminative performance of baseline ventricular dyssynchrony parameters for predicting response to CRT was evaluated using receiver operating characteristic (ROC) curve analysis. The area under the curve (AUC) with 95% confidence intervals (CI) was calculated for each parameter. Optimal cutoff values were identified using the Youden index, and corresponding sensitivity and specificity were reported.

**Predictive Modeling:**

A binary logistic regression model was constructed to determine predictive factors associated with a positive response to CRT, obtaining ORs and CIs to establish the association of baseline characteristics with CRT response. Given the small sample size and to minimize the risk of overfitting, only two basal variables were considered for analysis: Global Longitudinal Strain (GLS) and basal Kapetanakis index.

**Correlation Analysis:**

The association between Δ6MWT (meters) and ΔKCCQ score (pre- and post-CRT) with the percentage of LVESV change at follow-up was evaluated using Spearman correlation coefficient. Results were illustrated using scatter plots.

A *p*-value < 0.05 was considered statistically significant for all analyses.

During the preparation of this manuscript, the authors used ChatGPT-5.1 for the purposes of minor improvements in language and writing. The authors have reviewed and edited the output and take full responsibility for the content of this publication.

## 3. Results

Information was collected from a total of 33 patients, of whom 10 were women (30%) and 23 were men (70%). The mean age was 60.4 ± 11.7 years. Regarding comorbidities, a prevalence of 48.5% (n = 16) of hypertension and 36.4% (n = 12) of type 2 diabetes mellitus was found. The etiology was ischemic in 48.5% (n = 16), followed by idiopathic, Chagas, valvular, and peripartum cardiomyopathy. Laboratory results highlighted an NT-proBNP value of 4409.9 (2195–6680 IQR) pg/mL. Electrocardiographic parameters related to synchrony showed a PR interval of 165 (160–200 IQR) ms and a QRS width of 175.2 ± 30 ms, reflecting significant AV and interventricular dyssynchrony. Regarding echocardiographic variables in the general population prior to CRT, a markedly reduced LVEF (22.3 ± 7.5%), an elevated pulmonary artery systolic pressure (37.5 (31–52 IQR)), and a ventriculo-arterial coupling index of 0.47 ± 0.21 were found. According to synchrony parameters, the difference in peak systolic velocity between the septal and lateral walls by tissue Doppler was 80.8 ± 48.1 ms, with 23 patients (70%) above 65 ms. Using M-mode to evaluate dyssynchrony between the septal and posterior walls, a mean of 200 ± 85 ms was found. The difference in pre-ejection periods of the outflow tracts showed a mean of 50.6 ± 27 ms, with 56.3% of the population above 50 ms. Regarding AV synchrony assessment, mitral filling was 50.6 ± 8.5%, with 54.5% of the population < 40%. Septal flash was present in 14 patients (42.4%), apical rocking in 17 (51.5%), and GLS showed a mean of −9.6 ± 3.0%. The Kapetanakis synchrony index had a mean of 8.5 ± 5.3%.

When comparing pre- and post-CRT variables in the overall population, a statistically significant difference was observed in NT-proBNP levels (*p* = 0.01), reduction in PR (*p* = 0.001) and QRS (*p* = 0.01), increase in LVEF (*p* = 0.01), decrease in LVESV (*p* = 0.03), improvement in GLS (*p* = 0.04), and improvement in AV synchrony (ventricular filling time % *p* = 0.04), as well as intraventricular synchrony with better septal–posterior wall contraction difference in M-mode and septal–lateral wall difference by tissue Doppler. Finally, an improvement in the Kapetanakis synchrony index was also observed. All of this demonstrates the beneficial response of device implantation at the biochemical, electrocardiographic, and echocardiographic levels (Table 1).

After classifying the population into groups according to response, there were 14 responders (42%) and the remaining 19 (58%) were non-responders. The analysis between these groups prior to CRT implantation showed no differences in age, sex, comorbidities, or etiology, indicating a homogeneous population. The only significant differences found were in GLS (non-responders –7.7 ± 2.4%/responders –12 ± 1.3%, *p* = 0.001) and the Kapetanakis index (non-responders 11.4 ± 5.3%/responders 4.4 ± 0.68%, *p* = 0.002), reflecting greater subclinical LV dysfunction and greater ventricular asynchrony in non-responders (Table 2).

Regarding the analysis of both groups after CRT implantation, we confirmed the greater benefit of CRT in the responder population. A marked improvement was observed in NT-proBNP, Fractional area change of the right ventricle (FACRV%), tricuspid annulus plane systolic excursion (TAPSE), LVEF%, intra-and-interventricular synchrony, GLS%, and Kapetanakis index (Table 3).

Regarding the KCCQ in the general population prior to CRT implantation, a score of 47.3 ± 17.5 was obtained, and after CRT it was 70.1 ± 7.1, reflecting a significant improvement in this variable following device implantation. When dividing the population according to response, we observed that prior to CRT implantation the score was similar in both groups, with no significant difference; however, after device implantation, a statistically significant improvement was observed in the KCCQ score in responders (82.4 ± 6.7 vs. 57.8 ± 11, *p* = 0.001). This indicates greater clinical benefit of CRT in patients with a positive response, demonstrated by an increase of more than 20 points in the questionnaire score (Table 4).

Regarding the 6MWT, in the total population prior to CRT implantation, 439.8 ± 98.9 mts were obtained, and after CRT, 457.7 ± 90.2 mts. This difference in post-CRT walking distance was not significant (*p* = 0.317). When analyzing these results based on CRT response, we observed a walking distance of 462.4 ± 97 mts in responders and 452.3 ± 88 mts in non-responders, with no significant differences (*p* = 0.08). This shows that the walking distance is not directly related to CRT, regardless of the type of response (Table 5).

As we mention before, GLS and the Kapetanakis index were the only ones that showed significant differences between responders and non-responders at baseline; therefore, these variables were selected for ROC curve analysis.

ROC curve analysis showed that baseline GLS had excellent discriminative ability for predicting response to CRT (AUC ~0.95). The optimal GLS cutoff was approximately −10.5%, providing the best balance between high sensitivity (90–95%) and good specificity (80–85%). ROC curve for Kapetanakis synchrony index showed and inverse association with CRT response, yielding an AUC of 0.88, with lower values being associated with higher likelihood of response (optimal cut-off 5.8%, sensitivity 90%, specificity 80%).

**Figure 1:**
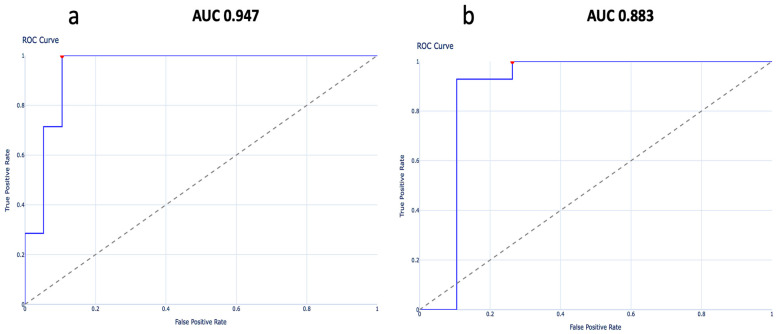
(**a**) Curve analysis of GLS for predicting response, (**b**) ROC curve analysis of Kapetanakis for predicting response.

These findings confirm that both GLS and the Kapetanakis index have high discriminatory power for identifying responders to CRT (Figure 1).

To reduce small-sample bias and potential overfitting of the model, binary logistic regression model was constructed using the interaction between response–GLS and response–Kapetanakis index.

Our results showed that for each unit increase in strain (most positive value) the probability of response decreases by 69% (95% IC 0.137 to 0.683, *p* = 0.004). Kapetanakis synchrony index was also significantly associated with the CRT response; for each unit increase in Kapetanakis index, the probability of response decreases by 54% (95% IC 0.23–0.93, *p* = 0.032) (Table 6).

Finally, we evaluated the correlation between clinical improvement (Δ6MWT distance and ΔKCCQ) and CRT response (% reduction in LVESV at follow-up) using Spearman’s rank correlation coefficient.

**Figure 2:**
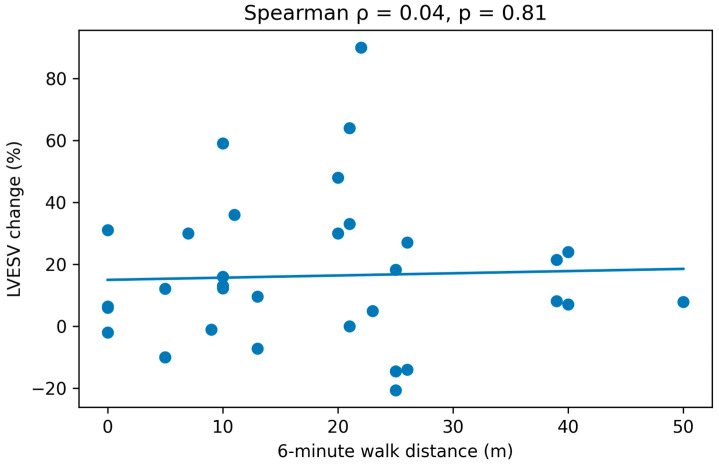
Scatter plot between LVESV change and Δ6MWT distance. A correlation coefficient (ρ) of 0.04 with *p* = 0.81 was observed.

No correlation was observed between the percentage reduction in LVESV (%) and Δ6MWT distance (mts), Rho spearman correlation coefficient (ρ) of 0.04 with *p* = 0.81 was obtained (Figure 2).

**Figure 3:**
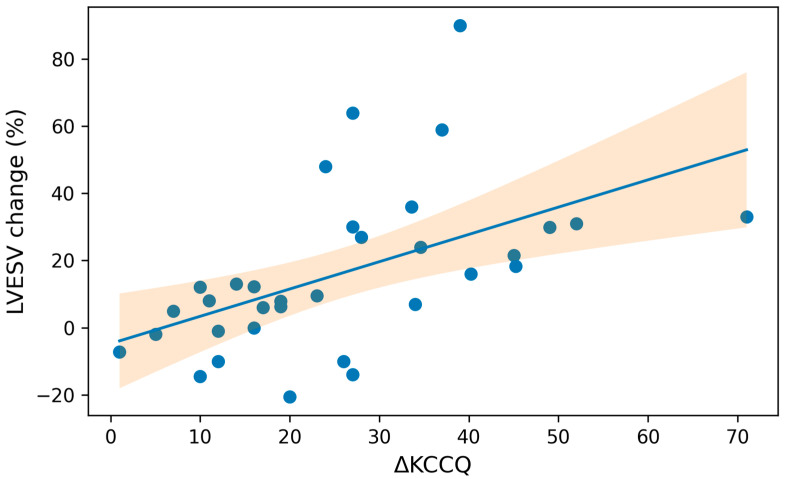
Scatter plot between degree of percentual reduction in LVESV (%) at follow-up and ΔKCCQ score.

Regarding QoL measured by KCCQ, the analysis showed a moderate–strong positive correlation between the degree of response to CRT and changes in KCCQ, Spearman correlation (ρ) of 0.663 with *p* = 0.001, which means that the greater the reduction in LVESV, the greater the improvement in QoL (Figure 3).

## 4. Discussion

This is the first prospective study in Mexico whose objective was to evaluate the benefit of CRT in patients with HF and reduced LVEF in a tertiary care hospital, as well as to identify predictive variables of a positive response. Beyond this, the study provides interesting information about patient characteristics, which contrasts with international data and could explain the low response rate in our population, points that will be addressed below.

In the demographic results, a mean age of 60.4 ± 11.7 years was observed, which is lower than that reported in previously published studies [17,18]. This is likely related to the high prevalence of chronic degenerative diseases in our population. Regarding the proportion of men and women, we observed a similar distribution in other studies, in which approximately 70% of this population is composed of men [15,19].

Our study also showed that ischemic etiology was present in 48.5% of the population, which is consistent with a retrospective analysis [11] carried out at our center. However, in most studies, ischemic etiology accounts for more than 54% [15]. This disparity may be explained by the fact that, although ischemic heart disease is the main cause of CHF, there are also other highly prevalent causes in Mexican population, such as Chagas cardiomyopathy, peripartum cardiomyopathy, and idiopathic cardiomyopathy. These conditions often occur at younger ages and with a better prognosis, which tends to prioritize them in resource allocation. It is worth remembering that non-ischemic etiology has been established as a predictor of positive response to CRT [20], which makes prioritizing these patients for device implantation.

Regarding baseline echocardiographic variables, we found an LVEF of 22.3%, which is similar to the retrospective study conducted at our center; however, it is lower compared to other studies where LVEF is around 35% [21]. In some others, LVEF was closer to 40%, and they concluded that patients with an LVEF of 35–45% showed a better response to CRT [22]. Likewise, there are studies where non-responder patients presented with more severely impaired LVEF compared to those with a positive response to CRT [23]. This information justifies the lower proportion of CRT responders in our study.

As mentioned previously, we use the reduction in LVESV to define the response to CRT because the evidence indicates that combining clinical, biochemical, and echocardiographic parameters does not improve the predictive value of this single parameter [24].

More recent studies and metanalysis have also adopted ≥ 15% reduction in LVESV as the definition of CRT response [25]. In our study, we observed a discrepancy in the percentage of responders, it was only 42%, whereas in others it has been reported above 50% [15,26].

When comparing baseline variables in responder vs. non-responder populations, only two variables were statistically different: GLS and the Kapetanakis index. This reflects greater myocardial damage in non-responder patients. The fact that the other variables showed no differences rules out the possibility that any of them acted as a bias in the level of response to CRT. In the studies by Aalen y cols. [26] and Rath y cols. [21], a significant difference was found in the proportion of ischemic patients, who were markedly predominant in the non-responder group, which probably indicates a bias effect on the device response.

Regarding post-CRT variables, we can state that the responder group showed a statistically significant improvement in NT-proBNP, suggesting subclinical improvement in congestive status. Tawfik y cols. corroborate this benefit, showing an approximate 50% reduction in NT-proBNP (1025 ± 363.1 to 594.9 ± 263.5, *p* = 0.0001) [22]. Other studies have even considered baseline NT-proBNP as a predictor of response [27]. Another parameter that showed clear improvement in responders vs. non-responders after CRT was LVEF, which increased by 10.4% in the responder population compared with only a modest 2.63% increase in non-responders. This benefit has been widely observed in both national and international studies [11].

Regarding interventricular dyssynchrony evaluated by the difference in pre-ejection periods, a more favorable response was observed in responder patients. Achilli et al. [28], in the SCART study, reported results from 133 patients in which this parameter predicted a positive response to CRT. Richardson et al., in the CARE-HF study, also demonstrated that a difference greater than 50 ms adds prognostic information in patients undergoing CRT [29]. However, the generalizability of these findings remains controversial; both trials were performed in highly specialized centers, thereby limiting their reproducibility and external validity. In the same line, the PROSPECT trial failed to demonstrate clinical utility, revealing that the ability of the echocardiographic parameters to predict clinical composite score response varied widely [15].

Intraventricular synchrony evaluated by the septal/posterior wall contraction difference in M-mode (ms) was significantly lower in responder patients, a finding supported by retrospective studies [11].

Another variable was GLS that improved significantly in the responder group. This confirms that responders tend to have both a more optimal baseline GLS and a more favorable delta post-CRT compared to non-responders. A similar result was obtained in the meta-analysis by Bazoukis et al. [30]. Also, in that study, the authors strengthen the central role of GLS as a tool for selecting candidates for CRT and suggest that improvements in this variable after CRT implantation could be used to define treatment response. In our study, we agree with this suggestion, as ROC curve analysis demonstrated that baseline GLS showed excellent discriminatory performance for predicting response to CRT, and unadjusted logistic regression analysis confirmed that baseline GLS was significantly associated with the response to CRT.

Furthermore, GLS has a close relationship with cardiovascular outcomes. Recent studies have found a significant association between a progressively worsening GLS at CRT implantation and prognostic outcomes on long-term follow-up [31,32].

As for the Kapetanakis index, previous studies have positioned this index as a predictor of adequate response to CRT, even in patients who do not meet all conventional criteria for device implantation [33]. Kapetanakis index has also been used for device optimization [34]. In our study, Kapetanakis index showed good discriminatory ability for the CRT response and established that lower values were associated with a higher likelihood of response, meaning that less dyssynchrony confers better response. There is previous evidence [35] confirming the high predictive value of the Kapetanakis index for NYHA class, LVEF, and reduction in LVESV (AUC 0.79, 0.86, and 0.66, respectively). In this study, patients with improvement LVESV (responders) showed a significant reduction in Kapetanakis index, and demonstrated that a cut-off value for Kapetanakis of 10.4% confers a sensitivity > 90% and a specificity of > 67% for all outcomes after CRT. It is important to note that, based on the Kapetanakis index, our population did not exhibit significant mechanical dyssynchrony (cut-off value of 10%), while in other studies this index exceeds 14% [35].

Regarding QoL assessed with the KCCQ, it was observed that overall patients had a score of 47 points, which is slightly higher compared to a recently published study [36]. In that study, the mean KCCQ score at 12 months was significantly higher compared to baseline, with a mean difference of –20.8261 (*p* < 0.001), indicating a substantial improvement in QoL after CRT. However, that study did not differentiate between responder and non-responder populations according to improvement in LVESV. Our study provides important information in this regard, showing that there is indeed an overall clinical benefit after CRT implantation; however, the greatest benefit was seen in responders, who increased their score by 34.9 points compared to baseline. In addition, our correlation analysis confirmed this relationship between the type of response (%) and the QoL. There is little evidence that has prospectively evaluated the relationship between QoL and response to CRT. In a retrospective study of the MADIT-CRT cohort [37], the population was divided into tertiles according to the KCCQ score, and it was concluded that after 12 months of follow-up, there was no significant difference in LVESV between these groups, which clearly contrasts with our study.

The 6MWT is a widely available and well-tolerated test for the assessment of the functional capacity of patients with HF. In our study, overall population showed an increase in the distance walked during the 6MWT after device implantation; however, this improvement was not significantly reflected after group classification. There is recent evidence of the favorable impact of CRT on walking distance when comparing the difference between pre- and post-CRT walking distance at 6, 9, and 12 months [38]. Interestingly, this study observed that the improvement in distance increased over time, showing continuous progress up to the 12-month [38]. Keeping this in mind, and considering our small sample size, it is possible that increasing the sample size or extending the follow-up period could reveal a significant benefit in this variable.

Although many variables have been widely validated as predictors of response, in our study, only GLS and Kapetanakis index provide evidence to support their use as predictive parameters, and we suggest considering them to improve patient selection and as prognostic parameters for patients undergoing CRT.

## 5. Conclusions

The present study provides valuable information regarding the profile of patients undergoing CRT in Mexico. Among the main differences compared with international studies, a younger age, diverse HF etiologies, and greater deterioration of ventricular function in our population stand out.

Despite the correct indication for CRT implantation, the positive response was only about 50%, which could be explained by the greater myocardial damage at the time of device implantation.

The benefit of CRT in the general population was clear, showing clinical, biochemical, and echocardiographic improvement (both in standard evaluation and synchrony parameters) at 6 months. This benefit was observed predominantly in the responder population.

A clear clinical benefit was confirmed assessed with the KCCQ. But this benefit was not observed in the 6MWT.

Only two parameters emerged as potential predictors of CRT response: the GLS and the Kapetanakis index.

## Figures and Tables

**Table 1:** Comparations of baseline biochemical, electrocardiographic, and echocardiographic characteristics of the overall population prior and after CRT.

Variable	Overall Population (n = 33)		
Sex (M/F) n, %	23/10 (70/30)		
Age (years)	60.4 ± 11.7		
Etiology, n (%)	Ischemic: 16 (48.5%)		
	Valvular 4 (12%)		
	Idiopathic 7 (21.5%)		
	Chagas 5 (15%)		
	Peripartum 1 (3%)		
Variable	Pre TRC (n = 33)	Post TRC (n = 33)	*p*
Na (mmol/L)	137 ± 4	138.5 ± 3.7	0.24
NT proBNP pg/mL	4400.9 (2195–6680)	1200 (855–3527)	0.01
PR ms	165 (160–200)	125 (120–160)	0.001
QRS ms	175.2 ± 30	129.4 ± 27.8	0.01
FACRV %	38.3 ± 10.4	39.6 ± 12.7	0.58
PASP (mmHg)	37.5 (31–52)	36.5 (30–50)	0.29
VA coupling	0.47 ± 0.21	0.78 ± 1.5	0.28
LVEF %	22.3 ± 7.5	28.4 ± 9.7	0.01
LA volume mL/m^2^	51.5 ± 17.7	50.8 ± 20	0.91
LVESV mL/m^2^	107.25 ± 48.1	96.7 ± 46	0.03
GLS	−9.6 ± 3	−10.6 ± 4.75	0.04
Dyssynchrony between septal/lateral wall by tissue Doppler, n (%) *	23 (70)	9 (27.3)	0.02
M-mode septal/posterior wall difference (ms)	200.36 ± 85	91 ± 47	0.01
Dyssynchrony by M-mode septal/posterior wall, n (%) *	30 (91)	5 (15.2)	0.02
Pre-ejection period difference (ms)	50 ± 27	42 ± 28	0.13
Dyssynchrony by pre-ejection period difference, n (%) *	18 (54.5)	12 (38.7)	0.87
Ventricular filling time < 40%, n (%)	18 (54.5)	9 (28.1)	0.04
Severe MR, n (%)	16 (48.5)	13 (40.6)	0.51
Apical Rocking, n (%)	17 (51.5)	16 (53.3)	0.28
Septal Flash, n (%)	14 (42.4)	10 (32.3)	0.53
Kapetanakis Index (%)	8.5 ± 5.3	6.8 ± 4.3	0.03

Abbreviations: FACRV—Fractional area change of the right ventricle; LA—left atrium; LVESV—left ventricular end-systolic volume; MR—mitral regurgitation; PASP—pulmonary artery systolic pressure; VA—Ventriculo-arterial; LVEF—Left ventricular ejection fraction; LV—Left ventricle; GLS—Global Longitudinal Strain. * Dyssynchrony was defined using the previously mentioned cutoff points for each variable in methods section.

**Table 2:** Baseline sociodemographic, clinical, biochemical, electrocardiographic, and echocardiographic characteristics in responder vs. non-responder patients prior to CRT.

Variable	Non-Responders (19)	Responders (14)	*p*
Sex (M/F) n, %	14/5 (73.7/26.3)	9/5 (64.3/35.7)	0.36
Age (years)	59.5 ± 2.6	61.7 ± 3.4	0.59
Hypertension, n (%)	9 (47.4)	7 (50)	0.59
Diabetes, n (%)	7 (36.8)	5 (35.7)	0.33
Ischemic etiology, n (%)	11 (57.9)	5 (35.7)	0.36
PR (ms)	183.3 ± 54.8	171.4 ± 27.4	0.14
QRS (ms)	178.9 ± 34	184.2 ± 17.8	0.47
Sodium mmol/L	137.6 ± 3.5	138.2 ± 3.6	0.67
NT Pro-BNP pg/mL	5538.9 ± 4000	3912 ± 2613	0.55
FACRV %	35.9 ± 12.8	39 ± 8.3	0.35
TAPSE mm	16.4 ± 4.1	17.3 ± 5.3	0.60
PASP (mmHg)	46.9 ± 17.1	36.65 ± 12	0.08
VA coupling	0.43 ± 0.21	0.52 ± 0.21	0.11
LA volume mL/m^2^	55.9 ± 18	46.3 ± 19	0.70
LVESV mL/m^2^	122 ± 40	125 ± 53	0.27
LVEF (%)	22.5 ± 7.4	21.9 ± 8	0.70
Dyssynchrony between septal/lateral wall by tissue Doppler, n (%)	13 (68.4)	10 (71.4)	0.33
Septal/posterior wall difference by M-mode (ms)	221 ± 101	171 ± 44	0.06
Dyssynchrony by M-mode septal/posterior wall, n (%)	17 (89.5)	13 (92.9)	0.77
Pre-ejection period difference (ms)	51 ± 26	49 ± 29	0.74
Dyssynchrony by pre-ejection period difference, n (%)	8 (42.1)	10 (71.4)	0.23
Severe MR, n (%)	9 (47.4)	7 (50)	0.28
Ventricular filling time < 40%, n (%)	5 (26.3)	13 (92.9)	0.51
Apical Rocking, n (%)	10 (52.6)	7 (50)	0.57
Septal Flash, n (%)	9 (47.4)	5 (35.7)	0.87
GLS	−7.7 ± 2.4	−12 ± 1.3	0.001
Kapetanakis Index (%)	11.4 ± 5.3	4.4 ± 0.68	0.002

Abbreviations: FACRV—Fractional area change of the right ventricle; LA—left atrium; LVESV—left ventricular end-systolic volume; MR—mitral regurgitation; PASP—pulmonary artery systolic pressure; VA—Ventriculo-arterial; LVEF—Left ventricular ejection fraction; LV—Left ventricle; GLS—Global Longitudinal Strain.

**Table 3:** Sociodemographic, clinical, biochemical, electrocardiographic, and echocardiographic characteristics in responder vs. non-responder patients after CRT.

Variable	Non-Responders (19)	Responders (14)	*p*
PR (mseg)	138 ± 40	133.8 ± 25.3	0.65
QRS (mseg)	134 ± 31	122.8 ± 22.3	0.36
Sodium mmol/L	139 ± 3.2	139.1 ± 3.2	0.37
NT Pro-BNP pg/mL	3332.53 ± 1910	1020 ± 549.7	0.01
FACRV %	34.4 ± 12.5	47.0 ± 10.2	0.01
TAPSE mm	16.1 ± 3.5	19.9 ± 4	0.04
PASP (mmHg)	42.6 ± 12.1	36.3 ± 13.4	0.34
VA coupling	0.49 ± 0.16	1.1 ± 2.1	0.29
LA volume mL/m^2^	57.6 ± 15	41.65 ± 23.3	0.11
LVESV mL/m^2^	107.1 ± 46.3	87.4 ± 44.5	0.03
LVEF (%)	25.13 ± 8.8	32.3 ± 10.0	0.04
Dyssynchrony between septal/lateral wall by tissue Doppler, n (%)	5 (26.3)	4 (28.6)	0.05
Septal/posterior wall difference by M-mode (ms)	124.2 ± 38	49.9 ± 19.5	0.00
Dyssynchrony by M-mode septal/posterior wall, n (%)	5 (26.3)	1 (7.1)	0.1
Pre-ejection period difference (ms)	54 ± 30.7	24.7 ± 14.7	0.01
Dyssynchrony by pre-ejection period difference, n (%)	11 ± 57.9	1 (7.1)	0.46
Severe MR, n (%)	9 (47.2)	1 (7.1)	0.11
Ventricular filling time < 40%, n (%)	2 (10.5)	1 (7.1)	0.26
Apical Rocking, n (%)	12 (63.2)	5 (35.7)	0.22
Septal Flash, n (%)	7 (36.8)	3 (21.4)	0.61
GLS	−7.1 ± 2.4	−15.4 ± 2.4	0.00
Kapetanakis Index (%)	9.6 ± 4.1	3.4 ± 0.65	0.00

Abbreviations: FACRV—Fractional area change of the right ventricle; LA—left atrium; LVESV—left ventricular end-systolic volume; MR—mitral regurgitation; PASP—pulmonary artery systolic pressure; VA—Ventriculo-arterial; LVEF—Left ventricular ejection fraction; LV—Left ventricle; GLS—Global Longitudinal Strain.

**Table 4:** Quality of life before and after CRT.

Variable	Non-Responders (19)	Responders (14)	*p*
Pre-CRT Score	47.26 ± 16.7	47.5 ± 20.98	0.92
Post-CRT Score	57.8 ± 11	82.4 ± 6.7	0.001

Abbreviations: CRT—cardiac resynchronization therapy.

**Table 5:** 6MWT (meters) according to response type before after CRT.

Variable	Non-Responders (19)	Responders (14)	*p*
Pre-CRT Walking Distance	443 ± 86	435 ± 78	0.4
Post-CRT Walking Distance	452.4 ± 97	462.4 ± 88	0.08

Abbreviations: CRT—cardiac resynchronization therapy.

**Table 6:** Regression analysis for predictors of CRT response.

Variable	β Coefficient	Odds Ratio (OR)	95% CI	*p* Value
Baseline GLS (%)	−1.183	0.31	0.14–0.68	0.004
Kapetanakis synchrony index (%)	−0.769	0.46	0.23–0.93	0.032

Abbreviations: CRT—cardiac resynchronization therapy.

## Data Availability

No new data were created or analyzed in this study.

## Abbreviations

The following abbreviations are used in this manuscript:

AV	Auriculo-ventricular
CHF	Cardiac hearth failure
CRT	Cardiac resynchronization therapy
LV	Left ventricle
LVEF	Left ventricular ejection fraction
LBBB	Left bundle branch block
KCCQ	Kansas City Cardiomyopathy questionnaire
LVESV	LV end-systolic volume
GLS	Global longitudinal strain
FACRV	Fractional area change of the right ventricle
6MWT	6 min walk test
QoL	Quality of Life
